# Targeting the Oncogenic p53 Mutants in Colorectal Cancer and Other Solid Tumors

**DOI:** 10.3390/ijms20235999

**Published:** 2019-11-28

**Authors:** Hui Li, Jinglin Zhang, Joanna Hung Man Tong, Anthony Wing Hung Chan, Jun Yu, Wei Kang, Ka Fai To

**Affiliations:** 1 Department of Anatomical and Cellular Pathology, State Key Laboratory of Translational Oncology, Prince of Wales Hospital, The Chinese University of Hong Kong, Hong Kong, China; haifei616@163.com (H.L.); jinglinzhang@cuhk.edu.hk (J.Z.); jtong@cuhk.edu.hk (J.H.M.T.); awh_chan@cuhk.edu.hk (A.W.H.C.); 2 Institute of Digestive Disease, State Key Laboratory of Digestive Disease, The Chinese University of Hong Kong, Hong Kong, China; junyu@cuhk.edu.hk; 3 Li Ka Shing Institute of Health Science, Sir Y.K. Pao Cancer Center, The Chinese University of Hong Kong, Hong Kong, China; 4 Department of Medicine and Therapeutics, The Chinese University of Hong Kong, Hong Kong, China

**Keywords:** TP53, p53 mutants, colorectal cancer, solid tumor

## Abstract

Colorectal cancer (CRC) is a kind of solid tumor and the third most common cancer type in the world. It is a heterogeneous disease characterized by genetic and epigenetic aberrations. The *TP53* mutation is the key step driving the transition from adenoma to adenocarcinoma. The functional roles of *TP53* mutation in tumor development have been comprehensively investigated. In CRC, *TP53* mutation was associated with poor prognosis and chemoresistance. A gain of function (GOF) of p53 mutants promotes cell proliferation, migration and invasion through multiple mechanisms. Restoring wild type p53 function, depleting p53 mutants, or intervention by targeting the oncogenic downstreams provides potential therapeutic strategies. In this review, we comprehensively summarize the GOF of p53 mutants in CRC progression as well as in some other solid tumors, and discuss the current strategies targeting p53 mutants in malignancies.

## 1. Introduction

Colorectal cancer (CRC) is the third most common cancer and the second leading cause of cancer deaths worldwide [1]. CRC is a heterogeneous disorder that possesses a variety of genomic and epigenetic aberrations. The loss of genomic stability drives the development of CRC by facilitating the acquisition of multiple tumor-associated mutations. These mutations control the capability of inhibiting tumor-suppressive genes and activating oncogenes, which have long been considered crucial events in CRC carcinogenesis. The earliest genetic event in colorectal tumorigenesis is activation of WNT signaling through the genetic disruption of *APC*. The *APC* somatic mutations rates increasingly with the disease progression, forming dysplastic aberrant crypt foci to adenomas and to sporadic carcinoma, with 5%, 30%–70% and 72%, respectively. It suggests that functional loss of *APC* is the initiating event in CRC [2,3,4]. The p53 pathway can be inhibited by mutation of *TP53*. This is the second key genetic step in CRC, which coincides with the transition from large adenomas into adenocarcinoma. *TP53* mutation occurs in 40%–50% of sporadic CRC [5]. The status of *TP53* mutation is correlated with progression and poor outcome of CRC. In recent years, some small molecule compounds have been intensively investigated for reactivation and restoration of p53 via different mechanisms. Some promising compounds are being tested in clinical trials and may be approved and employed for the treatment of CRC patients in the near future.

## 2. The Introduction of *TP53* and Tumor Suppressive Role of p53

### 2.1. The Finding of TP53

In 1979, the p53 protein was first identified. Its discovery was a product of research into viral etiology and the immunology of cancer. In 1979, two groups simultaneously reported similar results about the existence of a protein of around 55 kDa that bound to large T antigen in various types of cancerous cells [6,7]. The p53 protein was named from its protein weighing 53 KDa. The protein was actually regarded as an oncogene at that time, as many tumors produce abundant levels of this protein—a phenomenon that was not observed in normal tissue, and ectopic expression of newly cloned *TP53* cDNA was shown to cooperate with oncogenic Ras to transform primary cells in culture [8,9,10]. However, it was soon recognized that p53 overexpression could transform cells and promote *in vivo* tumor growth. This discrepant finding can be attributed to the use of different mutated versions of p53, which originally derived from tumor cells [11,12]. In 1989, the first murine wild type *TP53* cDNA was cloned and it was found that there was an absence of oncogenic activity [13]. The reports of the anti-proliferative property of wild type p53 and the demonstration of inactivating mutations in colorectal cancer eventually changed the recognition of *TP53* to a tumor suppressor gene [14,15].

### 2.2. The Functional Role of Wild Type p53

The tumor suppressor gene *TP53* is located on the short arm of chromosome 17 (17p13.1), and p53 is a modular protein with 393 amino acids harboring four functional domains. The N-terminal domain or transactivation domain (NTD or TA) possesses an acidic N-terminal transactivation domain (1–42) and a proline-rich domain (40–92). The proline-rich domain contains a second transactivation domain, which is essential for binding to transcription factors and regulators of p53 activity [16]. The centrally located sequence-specific DNA-binding domain (DBD) (101–306) allows the binding to DNA [17]. Followed by an oligomerization domain (307–305), which contains a nuclear export signal involved in the tetramerization of p53, and a basic C-terminal regulatory domain (356–393) includes three nuclear localization signals, which are relevant for the post-translational modifications [18,19]. p53 is a short-lived protein with a half-life of 6–20 minutes. The amount of p53 protein in cells is determined mainly by the rate at which it is degraded. In normal cells, the ubiquitin-mediated proteolysis is the main process for p53 degradation, the MDM2 (murine/human double minute 2) protein is one of the enzymes involved in labelling p53 with ubiquitin. This process is a feedback loop. Once activated, p53 upregulates its negative regulator MDM2. Then, MDM2 binds with p53 to regulate the ubiquitination of p53, which leads to its degradation and helps to maintain low expression of wild type p53 [20]. In the presence of cellular stress, the p53 network is activated. One of the effects of p53 activation is to regulate the cell-division cycle. The cyclin-dependent kinases (CDKs) and cyclins are key regulators which control the cell cycle process. p21 (WAF1) is one member of the CDK inhibitors which take part in the inhibition of transition from G1 to S phase and G2 to M phase. *p21* is a downstream target gene of p53 and it has been shown to take part in the p53-induced cell cycle arrest, along with other p53 target genes including retinoblastoma protein (*Rb*), *GADD45*, *14-3-3σ*, and *cRRIMA-1^MET^* [21]. The Bax protein is one member of the Bcl-2 protein family, which can be activated by p53 to induce apoptosis [22]. Besides, Noxa and PUMA were found to be directly activated by p53 and their high expression can induce apoptosis [23]. p53 induces the expression of some DRs (death receptors), such as Fas, DR5 and PIDD, which form death-inducing signally complex with caspase-8 [24]. p53 expression may also induce death by direct stimulating mitochondria to produce an excess of highly toxic reactive oxygen species. Cellular senescence can be activated by p53. p16 and PML have been reported to take part in this activity, as well as p21 [25]. p53 also plays an important role in genome integrity, and in the regulation of cell metabolism [26,27]. Moreover, p53 promotes GLS2 transcription, leads to glutamine metabolism and reduces Reactive Oxygen Species (ROS) production [28]. p53 regulates nucleic acid biosynthesis. It can promote lipid biosynthesis by inhibiting SREBP-1 and G6PD, or inhibits lipogenesis by activating SIRT1, Aromatase, Acad11, Lipin1, MCD, and DHRS3 [29,30]. p53 plays a role in inhibiting cell migration and invasion through blocking filopodia formation, fibronectin formation, cellular expansion and polarization, which is mediated by suppressing Rac, RhoA, and Cdc42 expression [31]. p53 exerts a suppressive role in angiogenesis by activating the expression of angiogenesis inhibitor TSP-1 as well [32,33].

## 3. *TP53* Mutation in CRC and Other Solid Tumors

### 3.1. TP53 Mutational Spectrum in CRC

*TP53* is among the five most frequently mutated genes in human cancer. The frequency of *TP53* mutations is highly variable between different cancer types. The *TP53* alteration data can be accessed from the IARC *TP53* Database (http://www-p53.iarc.fr/) [34], which compiles all *TP53* mutations reported in human cancers, updated to August 2018. In this database, the *TP53* mutation frequency ranges from 14.4% in endocrine gland tumor to 43.28% in CRC. More than 96% of *TP53* mutations are mainly located in exons 5, 6, 7, 8 and 4, and 93% of *TP53* mutations cluster are between codon 100 and 300, which is regarded as the DNA-binding domain. Among them, codon 248, 273, 175, 245, 282 and 249 are the top six mutated codons, with 6.79%, 6.55%, 4.8%, 3.12%, 2.59% and 2.59% mutational rates, respectively. The transversion of GC > AT at CpG is the highest in all the transversion types. *TP53* mutations are associated with lymphatic invasion in proximal CRC and show a significant correlation with both lymphatic and vascular invasion in distal CRC. CRC patients with mutant p53 appear more chemo-resistance and have a poorer prognosis than those with wild-type p53.

### 3.2. The Mechanisms of p53 Mutants with Gain of Fucntion (GOF)

**Figure 1:**
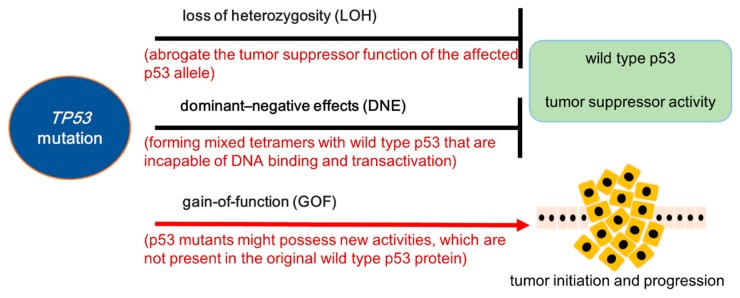
The consequences of somatic *TP53* mutations in tumorigenesis. The outcomes of p53 mutants are loss of wild type function and gain of new function. The LOH (loss of heterozygosity) and DNE (dominant-negative effect) are the two major main mechanisms to abrogate the tumor suppressor function of wild type p53. In some cancer cases with *TP53* mutations, the GOF (gain-of-function) of p53 mutants are empowered with kinds of oncogenic potentials, which promote cancer initiation and progression.

There are two major genetic mechanisms about the p53 locus alteration. One is the deletion of *TP53* gene, which is mainly induced by transacting mutation, deletion, insertion or frameshift substitution. Another is missense mutations, which has been found to attenuate p53 function. The outcomes of p53 mutants are loss of tumor suppressor activity or GOF to support tumor progression. Among cancers with *TP53* missense mutations, about 60% show concomitant deletion of the other allele. This deletion is termed as loss of heterozygosity (LOH) which results in abrogate the tumor suppressor function of the affected *TP53* allele [35]. Around 40% of cases with p53 mutants keep a wild-type *TP53* allele. In these cases, it has been shown that the dominant negative effects (DNE) of p53 mutants play a repressing role on the wild type p53 protein (Figure 1).

**Figure 2:**
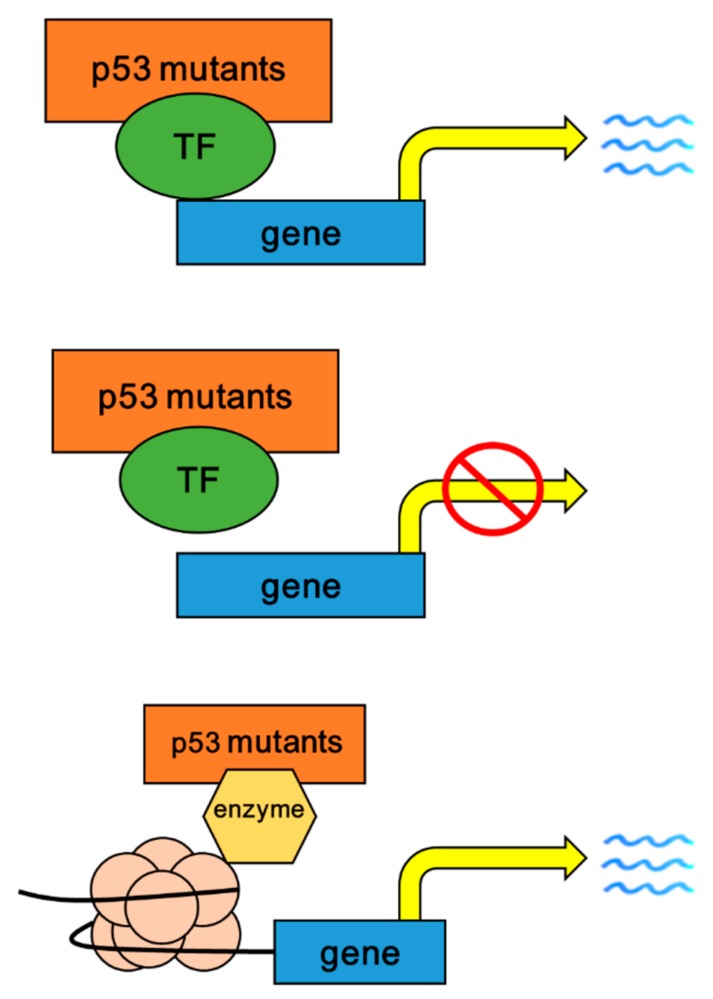
The multiple mechanisms of p53 mutants with GOF in CRC and other solid tumors. Upper, p53 mutants bind to TF (transcription factor) and activates target gene expression, such as c-Myc and Bcl-XL. Middle, p53 mutants bind to TFs (p63 and p73) and subvert their binding affinities to targeted promoters, which suppresses target gene expression. Lower, p53 mutants bind to and activate chromatin modifying enzymes to remodel the chromatin and promote target gene transactivation.

The p53 mutants retain the ability to form a protein–tetramer complex. This kind of complex is called a heterodimer complex as it is a mixture of mutated and wild type proteins, which suppress p53 wild-type function by inhibiting its normal DNA binding activity [36]. While losing tumor suppressive function, p53 mutants can gain new oncogenic functions to promoting cell transformation, tumor progression, metastasis, and chemo-resistance [37]. The mechanisms of p53 mutant GOF have been well studied. Plenty of studies have examined the binding affinity of different p53 mutants to known transcription factors (TFs). In colon cancer, the mutant R248W binds to Mre11, while R273H binds to NF-Y, Mre11, or YAP1 in cell lines HT29, SW620, SW480, respectively [38,39]. The p53 mutants R175H, R248W, R273H, Y220C, E258V, R110P and R282W have been shown to interact with p63 and p73, which prevents their binding to targeted promoters and inhibits target gene expression [37]. A common set of TFs such as ETS1/2, E2F1, NF-Kb and SMADs may be bound by different p53 mutants and activate their target genes to impart GOF activities. Likewise, p53 mutants promote the expression of c-Myc and Bcl-XL, which enhances cell growth [40]. Meanwhile, the binding of p53 mutants with p63 and p73 subverts their target gene activation, which promotes cell motility, invasion and metastasis [41,42]. In addition, a single p53 mutant variant may interact with multiple TFs, which activates a number of target genes. Collectively, the interaction between p53 mutants with other TFs has been recognized as main mechanism to mediate and amplify the GOF of p53 mutants. Also, p53 mutants increase chromatin accessibility and enhance gene expression within spans of accessible chromatin (Figure 2).

### 3.3. Oncogenic Roles of p53 Mutants with GOF

The concept that p53 mutants may exert GOF activity was previously demonstrated by introducing the p53 mutant protein into p53 null cells, which promoted cell growth and tumorigenic potential [43,44,45]. Since this discovery, studies have shown many GOFs in numerous cell lines with a variety of p53 mutations. These GOF activities include promoting tumorigenesis, activating tumor invasion and migration, and driving chemo-resistance. Recent findings are summarized in Table 1.

#### 3.3.1. Enhancing Cell Proliferation and Colony Formation

p53 mutants play an important role in sustaining the proliferation and evasion of growth suppression in tumorigenesis. In glioblastoma, mutant p53 R273H forms a complex with CBP and NFY, then activates the oncogenes c-Myc and Bcl-XL, thus promoting cancer cell proliferation, colony formation, invasion and survival [40]. The p53 mutants R280K and R282W were found to suppress KLF17, which is a negative regulator for breast cancer metastasis. The suppression of KLF17 by p53 mutant enhanced cell growth, migration and invasion [46]. A novel mutant p53 R273H/miR-27a/EGFR pathway is discovered that play important roles in facilitating cancer cell proliferation, colony formation and tumorigenesis [47]. In lung cancer, several p53 mutants (R175H, R273H, R273C, D281G and R267P) are shown to increase Axl expression, leading to cell growth and tumorigenesis [48]. p53 mutants also promote tumor growth in a xenograft mouse model [49]. p53 mutants are identified along with their binding partner, ETS2, to regulate the expression of numerous nucleotide metabolism genes (NMG), which sustain proliferation and invasion [50]. Enhanced proliferation is also seen upon the activation of the REG-γ proteasome pathway by p53 mutants [51,52].

Multiple factors are involved in the p53 mutants mediated colony formation. p53 mutant R273H attenuates the activation of NRF2, directly resulting in functional loss of NRF2 in ROS detoxification. In cells expressing p53 mutant, the high level of ROS fails to induce growth arrest but promotes cancer cell growth and colony formation [53]. TopBP1 is found to promote p53 mutants and p300 recruitment to NF-Y target gene promoters, thus inhibiting the transcriptional activities of p63/p73. TopBP1 is believed to mediate cancer cell growth and chemotherapy resistance caused by p53 mutants [54]. HSF1 can also affect clonogenic growth in a p53 context dependent manner. In p53 mutant existence, HSF1 significantly increased cells clonogenic growth [55]. p53 mutants increase GRO1 expression, which promotes cancer cell growth and colony formation. While GRO1 knockdown inhibits cell growth and abrogates p53 mutant GOF in SW480 cells [56].

#### 3.3.2. Promoting Migration, Invasion and Metastasis

Cells expressing p53 mutants R175H, R273H and D281G show increased migration, with elevated expression of CXCL5, CXCL8 and CLCX12. The inhibition of CXCL5 by RNAi suppressed cell migration [57]. In breast cancer, Pin1 enhances tumorigenesis in a p53 mutant knocking mouse model and it amplifies p53 mutant pro-migration function in vitro. Patients with high expression of Pin1 with p53 mutants show the worst overall survival [58]. The target genes regulated by p53 mutants include c-Myc, Bcl-XL, KLF17, Axl, Sharp1, Cyclin G2 and other oncogenes regulated by let-7i [40,41,46,48,59].

The role of p53 mutant GOF in invasion and metastasis is one of the most well-studied points. The p53 mutant R175H and R273H are found to promote invasive behavior in cancer and normal cells in vitro and in vivo. This is mediated by AKT signaling activation driven by p53 mutants. RCP-dependent recycling of integrin and EGFR are involved in this process [60]. Alternatively, p53 mutants promote cancer invasion through inhibition of Dicer function by direct downregulation of the TAp63 dependent transcriptional activation of Dicer or independent manner [61]. The p53 mutants are also found to enhance MET recycling to promote cell scattering and invasion in TAp63 dependent and independent mechanisms [62]. p53 mutants could interact with and inhibit TAp63 to regulate genes that increase invasion through TGF-β signaling [41]. p53 mutants were observed to promote cancer cell invasion by attenuating and cooperating with the TGF-β pathway by targeting Smad3 [42]. miRNA let-7i is found to be a direct target of p53 mutant/p63 complex, which affects cancer cell invasion; migration by regulating a network of oncogenes including E2F5, LIN28B; and NRAS [59].

Various other TFs play roles in p53 mutant-driven invasion and metastasis. It has been shown that p53 mutants enhance pancreatic cancer cell metastasis by modulating p73 and through an interaction with NF-Y. p53 mutants induce PDGFR-β expression through inhibition of p73/NF-Y complex. Blocking of PDGFR-β prevents cancer cell invasion and metastasis formation both in vitro and in vivo [63]. In glioblastoma, a PTEN-dependent p53 mutant/CBP/NF-Y complex transcriptionally activates the oncogenes c-Myc and Bcl-XL, which results in enhanced cell proliferation, colony formation and invasion [40]. Through interacting with SREBPs, p53 mutants regulate the mevalonate pathway, leading to increased proliferation, invasion and metastasis [64]. In breast cancer patients, the high expression of genes in mevalonate pathway is associated with particularly poor survival. EGR1 is another TF regulated by p53 mutants, which activates Myo10 via MAPK/ERK signaling. Increased Myo10 promotes breast cancer invasion and metastasis [65]. Other genes directly regulated by p53 mutant, such as KLF17 and REG-γ, also participate in breast and endometrial cancer cell invasion, respectively [46,51].

In intestinal tumors, a loss of wild-type p53 alone might facilitate the invasion of benign tumors, although it is insufficient to drive the malignant process [66]. A previous study provides evidence in an *APC^Δ716^* mouse model suggesting the nuclear enrichment of mutant p53^R270H^ leads to tumor gland formation. This drives malignant progression and enhances the acquired invasiveness. Additionally, the mutant p53^R270H^ upregulates a subset of genes associated with the activation of inflammatory and innate immune pathways. By that means, nuclear accumulation of p53^R270H^ not only increases stemness in intestinal tumor cells by activating NF-κB pathway and Wnt signaling, but also remodels the microenvironment to accommodate the invasive properties of the tumor cells [67]. Cooperation of p53 mutants with other driver mutations and oncogenic pathways plays significant role during the multistep CRC tumorigenesis; thus, targeting p53 mutants might serve as an ideal strategy to inhibit CRC progression.

#### 3.3.3. Inducing Angiogenesis

p53 mutants play an active role in promoting angiogenesis in vivo. The xenografts with p53 mutant expression demonstrate increased number of vessels compared with wild type p53 expressing tumors. This stimulation is depending on increased ROS level, which leads to upregulation of HIF1/VEGF pathway [68]. A significant correlation between high p53 mutant expression and VEGF is also found in breast cancers and gastrointestinal cancers [69,70]. The transcriptional axis of p53 mutants, E2F1 and ID4 are found to serve as a new molecular mechanism in promoting tumor angiogenesis. The complex of p53 mutants and E2F1 positively controls ID4 expression. The abundant ID4 activates pro-angiogenic factor IL8 and GROα, which supports the promotion of angiogenic potential of p53 mutants [71].

#### 3.3.4. Inducing Chromatin Remodeling

In breast cancer, Pfister et al. discovered that VEGFR2 is a p53 mutant transcriptional target [72]. The binding of p53 mutants on the promoter region of VEGFR2 makes it open to benefit the binding of p53 mutant with the chromatin remodeling complex SWI/SNF. The SWI/SNF complex plays a an important role in maintaining VEGFR2 promoter remodeling. The absence of SWI/SNF leads to increased nucleosome occupancy, which decreases the binding of p53 mutants on VEGFR2 promoter and suppresses VEGFR2 expression [72].

p53 mutant was found to be involved in the transcription of the MLL1 and MLL2 histone methyltransferases and the MOZ histone acetyltransferase. This results in global changes in histone modifications, which enhances the activities of p53 mutant GOF. MLL1 and MLL2 are members of the SET family of histone methyltransferase enzymes. They act as parts of large complexes to regulate gene expression by attaching methyl groups to a H3K4 protein. The H3K4 methylation promotes expression of the gene packaged around the histones. The binding of p53 mutant with MLL1 and MLL2 leading to genome-wide histone methylation, which finally enhances cancer cell growth in vitro. The p53 mutant activates the transcription of gene MOZ, which encodes an enzyme that plays a role in shifting the acetyl group to H3K9. The level of H3K9 acetylation depends on p53 mutant expression, which confirmed the idea that p53 mutant affects histone modification. These results suggest new possibilities for cancer therapy by targeting chromatin modification for cancers driven by p53 mutants. For example, knocking down MLL1 markedly decreased tumor cell growth, and pharmacological inhibition of the MLL1 methyltransferase complex inhibited proliferation of cancer cells carrying p53 mutations, but did not affect those without p53 mutants [73].

p53 mutants might alter the enhancer landscape of tumor cells under the condition of chronic immune activation [74]. The enhancer specific p53 mutant binding events are correlated with RNAPII recruitment. p53 mutants modulate the TNF-α inducible activation of enhancers through regulation of RNAPII recruitment. In terms of the regulation of enhancer associated alterations, the patterns between p53 mutants and wild type were quite different. The expression of eRNA and mRNA of MMP9 and CCL2 is higher in tumors with *TP53* mutation compared with tumors expressing wild-type p53. The findings support that the majority of p53 mutants are mis-localized to other transcriptional regions and interact with abnormal transcriptional regulators, while wild-type p53 is often recruited to typical sites. The p53 mutants regulate chromatin modification and this provides an opportunity of targeting chromatin modifying activities. JQ-1, a BRD4 inhibitor, can decrease the gene expression regulated by super enhancers [75]. BRD4 is a member of bromodomain and extraterminal domain (BET) proteins family. BET proteins recognize acetylated lysine residues on histone tails, recruit chromatin-modifying enzymes and transcription factors. BRD4 thereby plays key roles at the interface between chromatin modification and transcriptional regulation. The combination of JQ1 with arsenic sulfide synergistically activates wild type p53 and inhibits c-Myc in gastric and colon cancer cells and xenografts model [76,77].

## 4. Targeting p53 Mutants in Tumorigenicity

**Figure 3:**
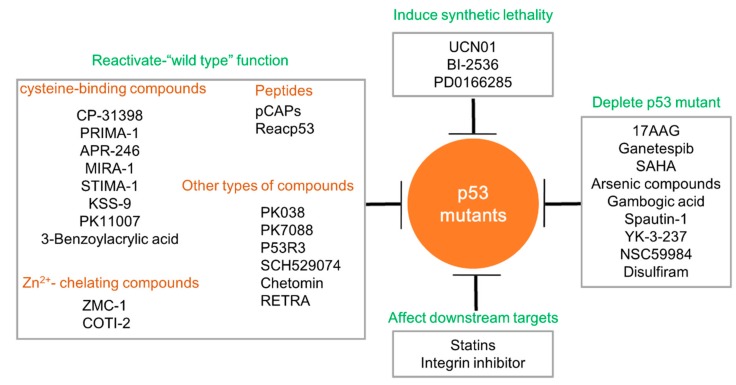
Strategies targeting p53 mutants with GOF. The strategies have been extensively undertaken to develop small molecular compounds that specifically target p53 mutants. The detailed mechanisms include restoring p53 wild type function, depleting p53 mutants, inducing synthetic lethality of p53 mutants and inhibiting the oncogenic downstream targets of p53 mutants. The cysteine-binding compounds, Zn2+-chelating compounds, peptides and other types’ compounds are employed to reactivate p53 wild type function, which are listed in the frame on the left. The compounds inducing p53 mutant depletion are listed in the right frame. The upper frame demonstrates the compounds which could induce synthetic lethality, including UCN01, BI-2536 and PD0166285. Statins and Integrin inhibitor have been reported to successfully inhibit p53 mutants’ downstream targets.

Much evidence shows that p53 mutants are stabilized in solid tumors and exert oncogenic GOF activities to promote cancer progression. The restoration of wild-type p53 function has been shown to be sufficient to induce rapid tumor regression in mice [82,83,84]. A larger number of studies have tried to exploit small molecule inhibitors targeting p53 mutants specifically. The main strategies include restoring the wild-type activity of p53 mutants, degrading p53 mutants, promoting synthetic lethality to p53 mutants, and quenching the related oncogenic p53 mutants’ downstream pathways (Figure 3).

### 4.1. Restoring the Function of Wild Type p53

#### 4.1.1. Cysteine-Binding Compounds

CP-31398 is the first compound which protects native wild-type p53 from a denatured conformation and restores the wild-type function of some p53 mutants. It was identified through a structure-based screening. In various tumor cell lines, CP-31398 has been found to promote p21 and MDM2 expression [85]. p21 is upregulated in Saos-2 cells with ectopic expression of p53 mutants V173A and R249S, after treatment with CP-31398 [86]. CP-31398 inhibits tumor growth in CRC and melanoma xenograft mouse model [87]. CP-31398 can refold newly synthesized p53 mutants, but is unable to refold already misfolded mutant p53 proteins. This compound also promotes DNA damage and apoptosis in skin carcinoma cell line with p53 R273H mutant [88].

PRIMA-1 is the most well studied small molecule, and its methylated analog PRIMA-1MET (also known as APR-246) has been tested in phase I/II clinical trial. PRIMA-1 was originally identified from a screening of small molecules that inhibit cell proliferation in the Saos-2 cell line with ectopic expression of p53 mutants R273H and R175H, compared with control Saos-2 p53 null cells [89]. PRIMA-1 can restore DNA contact mutants, as well as structural p53 mutants. In the test, it has been found to rescue specific DNA binding to 13 of 14 p53 mutants, which leads to restoration of wild-type conformation and causes apoptosis in tumor cells [89]. PRIMA-1 and PRIMA-1MET are found to inhibit the proliferation of tumor cell lines with p53 mutations in vitro and quench the xenograft formation in animal models [90,91,92]. PRIMA-1 and PRIMA-1MET can activate wild type p53 target genes, including p21, MDM2, Noxa, Puma, BAX, GAD45 and miRNA-34a, which enhance cell death [90,91,92]. In addition, PRIMA-1MET was found to exert proapoptotic activity by conversion to more active molecule methylene quinuclidinone (MQ), which leads to an oxidative environment [93]. In phase I/IIa clinical trial, PRIMA-1MET have been confirmed to reduce bone marrow blasts in a patient with AML and reduce tumor size in a patient with non-Hodgkin lymphoma. A phase II clinical trial that evaluated the effects of a PRIMA-1MAT combination with carboplatin and pegylated doxorubicin is ongoing in high grade serous ovarian cancer patients with p53 mutants [94]. Phase Ib/II studies have begun, including a study of PRIMA-1MET in combination with cytosine analogue azacitidine in myelodysplastic syndrome and a study of PRIMA-1MET in combination with cisplatin and 5-FU in esophageal cancer [95,96]. Several other Cysteine-binding compounds have been also reported to restore wild type function of p53 mutants, including MIRA-1, STIMA-1, KSS-9, PK11007 and 3-Benzoylacrylic acid [97,98,99,100,101,102].

#### 4.1.2. Zn^2+^-Chelating Compounds

Zinc is required for the proper folding of wild type p53, while the proper protein folding and function is prevented in p53 mutants lack of zinc binding ability. The addition of zinc to cells and tumors in a mice model with p53 mutations R175H and R273H has been found to restore p53 mutants’ DNA binding ability and prevent tumor progression [103]. Zinc metallochaperone-1 (ZMC-1), also known as NSC319726, has been identified to promote the binding of zinc with p53 mutants that restore the proper folding and transcriptional activity [104]. This was discovered from a screening with the NCI60 tumor cell line panel. The results showed that it is specifically toxic to cells with p53 R175H mutation. ZMC-1 repairs the wild-type-like conformation of p53 mutants and generates ROS to activate p53 downstream target genes including p21, PUMA and MDM2. Importantly, ZMC-1 induces greater toxicity to tumors in specific p53 R172H (equivalent to human R175H) mice and suppresses xenograft formation of cancer cells with p53 R175H mutant [104]. In addition, other zinc binding p53 mutations C176, C242, C238, H179 and M237 may been functionally reactivated by ZMC-1. COTI-2 is another Zn^2+^-chelating compound that has been reported to restore the folding and function of p53 mutants. It inhibits the PI3K–AKT pathway, induces cell death, and prevents xenograft growth in mice, carrying both p53 mutants and wild-type [105]. The exact mechanism is still not comprehensively understood. This kind of compound is now being tested in a phase I clinical trial in gynecological and head and neck cancers [106].

#### 4.1.3. Peptides

Several peptides have been found to reactivate p53, which suppresses tumor growth in mouse xenograft models of breast, colon and ovarian cancer. A series of peptides were identified to convert p53 mutant conformation to the wild-type-like form, activate p53 targets, and induce apoptosis in tumor cells [107]. Reacp53 is found to block p53 mutant aggregation, and rescues p53 wild-type properties which induce cell cycle arrest and promote apoptosis in ovarian cancer cells [108].

#### 4.1.4. Other Types of Compounds

The compounds PK083 and PK7088 can bind to p53 Y220C specific surface cavity, resulting in the refolding of the wild-type conformation [109,110]. P53R3 has been shown to restore the DNA-binding ability of p53 mutants 175H, R273H and M237. Further, it can increase the expression of p21, MDM2, BAX, PUMA, GADD45 and PIG3, and exert the p53 dependent anti-proliferation activity [111]. Another compound targeting p53 mutants is SCH529074. It can bind to p53 mutants’ core domain, increases p53 target genes’ expression, and inhibit p53 ubiquitination by MDM2 [112]. Chetomin, which binds to the heat shock protein 40 and promotes its interaction with p53 R175H mutant, suppresses growth of xenograft tumor in mice [113]. RETRA, which specifically disrupts the complex of p53 and p73, inhibits p53 R273H mutated xenograft growth [114].

### 4.2. Depleting Mutated p53 Proteins

In vitro experiments demonstrated that knocking down p53 mutants by siRNA suppresses cancer cell growth. An in vivo genetic ablation of p53 mutants induced by Tamoxifen effectively antagonized tumor invasiveness. It proves that GOF p53 mutants are feasible druggable targets in CRC [115]. Thus, small molecules that specifically deplete p53 mutants are promising for cancer therapy. 17AAG, an inhibitor of HSP90, has been reported to degrade p53 mutants R175H, R273H, L194F and R280K and inhibit the tumor cell growth, which carries p53 mutations [116]. Ganetespib, another HSP90 inhibitor, is currently being tested in a clinical trial of lung cancer and it has been shown to be over 50-fold more potent than 17AAG in depleting p53 mutants [49]. The histone deacetylase inhibitor SAHA degrades p53 mutants by inhibiting HDAC6, and the disrupted HDAC6-HSP90-p53 mutant axis can lead to mutant p53 ubiquitination [117]. The arsenic trioxide increases transcripts of Pirh2 and induces proteasomal dependent degradation of several p53 mutants, including R175H, R248W, H179Y/R282W and R273H [118,119]. Gambogic Acid, spautin-1, YK-3-237, NSC59984 and Disulfiram are also found to degrade p53 mutants [120,121,122,123,124,125].

### 4.3. Inducing Synthetic Lethality to p53 Mutants

Inducing synthetic lethality of p53 mutant in tumors is another strategy for therapy. UCN01, an inhibitor of protein kinase C and a potent blocker of G2/M checkpoint, has been shown to increase the irradiation cytotoxicity to CA46 cells with p53 R248Q and HT29 cells with p53 R273H [126]. The BI-2536 and PD0166285 also have been observed to lead synthetic lethality to p53 mutants through hindering G2/M transition. These two compounds can increase the cytotoxic effect of ionizing radiation in DLD-1 p53 S241F and HCT116 p53 R248W cell lines, but not in a cell line with wild-type p53 [127,128].

### 4.4. Targeting the Oncogenic Downstreams of p53 Mutants

In order to suppress the oncogenic property of p53 mutants, another effective way is to disrupt tumor-promoting signaling activated by mutant p53. The 3-hydroxy-3-methylglutaryl coenzyme A (HMG-CoA) reductase regulates prenylation/lipidation of proteins. Prenylation plays a role in cellular adhesion, migration, and proliferation signaling through facilitating attachment of target proteins to cell membranes. Statins play an important role in suppressing HMG-CoA reductase, and have been applied in patients with hypercholesterolemia disease. In breast cancer cells, p53 mutant binds to and activates SREBP, resulting in activation of prenylated proteins. Thus, statins inhibit breast tumor growth through suppressing p53 mutants and protein prenylation [64]. Moreover, the Rho GTPases’ prenylation promotes nuclear localization and activation of the YAP/TAZ. Statins suppress Rho GTPase prenylation and YAP/TAZ activation, thus preventing tumor development carrying p53 mutants [129].

The main compounds potentially targeting p53 mutant are listed in Table 2.

## 5. Conclusions and Future Directions

Although many studies have explored the mechanisms of p53 mutants with GOF, the mechanisms have not been fully elucidated. Thus, targeted therapy on p53 mutants has been regarded as one of the hardest medical issues. More importantly, CRC is a heterogeneous disease with high recurrence rates and it is hard to achieve a better therapeutic effect by solely targeting p53 mutants. We are glad to see that several clinical trials targeting p53 mutants get satisfied therapeutic effects or combinational administration of p53 mutant inhibitors with other small molecules which exert a synergetic effect on inhibiting tumor growth. However, several urgent issues have to be addressed in the coming studies. First, as a transcription factor, p53 mutants bind with novel transcriptional regions and it is imperative to perform ChIP-seq on different p53 mutants to reveal the specific or common downstream targets or pathways. Second, the involvement of p53 mutants in histone modification, especially the relationship with super enhancer, has gradually become a hot topic in research. Thus, integrative analysis of p53-mutant ChIP-seq with H3K4me3/H3K27ac ChIP-seq needs to be performed to reveal novel epigenetically regulated targets. Last, but not least, stratifying the primary CRC samples and cell lines based on the p53 related genetic and epigenetic background will be performed for the accurate administration of small molecules in the wet-lab research and clinical trials. With the increased knowledge of p53 mutants, more and more advanced intervention strategies will be developed in the precision medicine era.

## Figures and Tables

**Table 1:** The promoting roles of p53 mutants with GOF in solid tumor cells.

Mutation Type	Cell Lines	Downstream Effectors	Refs
Promoting cell proliferation			
R273H	U373/SNB19	*c-Myc/Bcl-XL*	[40]
R280K/R282W	MDA-MB-231/MDA-MB-1386	*KLF17*	[46]
R273H	H1299	*miR-27a/EGFR*	[47]
R273H/R175H/D281G	H1299	*Axl*	[48]
R273C/R267P	H1048/H1437		
R238Q/R172H	-	*HSP90/HDAC6*	[49]
R249S/R273L/R280K	BT549/HCC38/MDAMB231	*EST2/NMGs*	[50]
R249S/R175H	MCF10a/H1299		
R248Q	HEC-1B	*REG-γ*	[51]
R175H	H1299/UMSCC-1	*REG-γ*	[52]
R280K/R282W/R273H	MDA-MB-231/MDA-MB-1386		
P278S/R267P	ABC1/H1437	*Axl*	[78]
R175H/R273H	H1299	*TopBP1*	[54]
R273C/R248Q/R175H	C33A/OVCAR-3/SKBr3		
R175H/R273H/R280K	SKBR3/HT29/MDA-MB468/MDA-MB231	*MAP2K3*	[79]
R280T	SWO-38	*GSK-3β/PTEN*	[80]
R280K/R273H	MDA-MB-231/MDA-468	*SREBPs*	[64]
Increasing colony formation ability			
R273H	U373/SNB19	*c-Myc/Bcl-XL*	[40]
R273H	H1299	*NRF2*	[53]
R175H/R273H	H1299	*TopBP1*	[54]
R273C/R248Q/R175H	C33A/OVCAR-3/SKBr3		
R273H	MCF10a	*HSF1*	[55]
R273H	H1299	*miR-27a/EGFR*	[47]
R175H	HCT116-/-	*GRO1*	[56]
R273H/P309S	SW480		
R248W	MIA-PaCa-2		
Increasing cell invasion and migration			
R273H	U373/SNB19	*c-Myc/Bcl-XL*	[40]
R280K/R282W	MDA-MB-231/MDA-MB-1386	*KLF17*	[46]
R249S/R273L/R280K	BT549/HCC38/MDAMB231	*EST2/NMGs*	[50]
R249S/R175H	MCF10a/H1299		
R248Q	HEC-1B	*REG-γ*	[51]
R175H/R273H	MCF10a/H1299	*RCP/integrin/EGFR*	[60]
R273H	H1299	*TAp63/Dicer*	[61]
R280K/R273H	MDA-MB-231/HT29/A431		
R175H/R273H	H1299	*TAp63/Met*	[62]
R175H	H1299	*TAp63/Sharp 1/Cyclin G2*	[41]
R175H	H1299	*Smad3*	[42]
R175H	H1299	*let-7i/E2F5/LIN28B/MYC*	[59]
R248W/R220C/H242R/H155P	Miapaca2/BXPC3/CFPAC/A2.1	*p73/NF-Y/PDGFR-beta*	[63]
R273H/R280K	SW620/H1975/MDA-MB-231		
R280K/R273H	MDA-MB-231/MDA-468	*SREBPs*	[64]
R280K	MDA-MB-231	*Myo10*	[65]
R175H/R273H/C135Y	HEC-50	*miR-130b/ZEB1*	[58]
R273H	U373/SNB19	*c-Myc/Bcl-XL*	[40]
R280K	MDA-MB-231	*KLF17*	[46]
R282W	MDA-MB-1386		
R273H/R175H/D281G	H1299	*Axl*	[48]
R273C/R267P	H1048/H1437		
R175H	H1299	*TAp63/Sharp 1/Cyclin G2*	[41]
R175H	H1299	*let-7i/E2F5/LIN28B/MYC*	[59]
R175H/R273H/D281G	H1299	*CXCL5/CXCL8/CXCL12*	[57]
R280K	MDA-MB-231	*Pin1*	[81]
Inducing angiogenesis			
R175H/R273H/R248W	HCT116-/-	*HIF1/VEGF-A*	[68]
R175H/R273H	H1299	*ID4/IL8/GRO-a*	[71]
R280K	MDA-MB-231		
Chromatin remodeling			
R248Q	HCC70	*MLL1/MLL2/MOZ*	[73]
R249S	BT-549		
R273H	MDA-MB-468		
R273H	SW480	*MMP9/CCL2/CYP24A1/CPA4*	[74]
R273H	MDA-468	*VEGFR2/SWI/SNF*	[72]

**Table 2:** The list of compounds targeting p53 mutants.

Reactivating the Wild Type p53 Function			
Compounds (Small Molecules)	Mechanisms	Clinical Trial (Cancers)	Refs.
Cysteine-binding compounds			
CP-31398	binds to the cysteine residues		[85,86,87,88]
PRIMA-1	converts to methylene quinuclidinone		[89,90,91,92]
APR-246	converts to methylene quinuclidinone	phase Ib/II (lymphoma, ovarian, esophageal)	[90,91,92,93,94]
MIRA-1	prevents unfolding of wild-type and mutant p53		[98]
STIMA-1	prevents unfolding of wild-type and mutant p53		[99]
KSS-9	prevents unfolding of wild-type and mutant p53		[100]
PK11007	binds p53 by nucleophilic aromatic substitution		[101]
3-Benzoylacrylic acid	binds p53 by Michael addition		[102]
Zn2+-chelating compounds			
ZMC-1	Zn^2+^ chelator		[104]
COTI-2	Zn^2+^ chelator	phase I (gynecological, head and neck cancer)	[105]
Peptides			
pCAPs	promote refolding		[107]
Reacp53	blocks aggregation		[108]
Other types of compounds			
PK083	restores wild-type conformation		[109]
PK7088	restores wild-type conformation		[105]
P53R3	restores DNA-binding ability		[106]
SCH529074	restores DNA-binding ability		[112]
Chetomin	promotes refolding		[113]
RETRA	disrupts mutant p53–p73 complexes		[114]
Depleting the GOF of p53 mutants			
17AAG	Hsp90 inhibitors		[116]
Ganetespib	Hsp90 inhibitors	phase III (lung cancer)	[49]
SAHA	HDAC inhibitors		[117]
Arsenic compounds	increases transcripts of Pirh2,and		[118]
	induces degradation of mutant p53		[119]
Gambogic acid	inhibits the mutant p53-Hsp90 complex		[121]
Spautin-1	induces mutant p53 degradation		[122]
YK-3-237	activates SIRT1 and deacetylate lysine 382		[123]
NSC59984	induces MDM2-mediated mutant p53 degradation		[124]
Disulfiram	induces p53 degradation		[125]
Inducing synthetic lethality			
UCN01	protein kinase C inhibitor		[126]
BI-2536	polo-like kinase 1 inhibitor		[127]
PD0166285	Wee1 kinase inhibitor		[128]
Blocking the oncogenic downstreams of p53 mutants			
Statins	HMG-CoA reductase inhibitor		[64]
	inhibits YAP/TAZ activation		[129]
